# Characterization of Influenza A(H1N1)pdm09 Viruses Isolated in the 2018–2019 and 2019–2020 Influenza Seasons in Japan

**DOI:** 10.3390/v15020535

**Published:** 2023-02-14

**Authors:** Takuma Soga, Calvin Duong, David Pattinson, Yuko Sakai-Tagawa, Akifumi Tokita, Naomi Izumida, Tamon Nishino, Haruhisa Hagiwara, Noriyuki Wada, Yumi Miyamoto, Haruo Kuroki, Yuka Hayashi, Masafumi Seki, Natsuko Kasuya, Michiko Koga, Eisuke Adachi, Kiyoko Iwatsuki-Horimoto, Hiroshi Yotsuyanagi, Seiya Yamayoshi, Yoshihiro Kawaoka

**Affiliations:** 1 Division of Virology, Institute of Medical Science, University of Tokyo, Tokyo 108-8639, Japan; 2 Department of Pathobiological Sciences, School of Veterinary Medicine, University of Wisconsin-Madison, Madison, WI 53706, USA; 3 Tokyo Pediatric Association Public Health Committee, Saitama 331-0815, Japan; 4 Clinic Bambini, Tokyo 108-0071, Japan; 5 Akebonocho Clinic, Tokyo 120-0023, Japan; 6 Alpaca Kids ENT Clinic, Tokyo 171-0052, Japan; 7 Hagiwara Clinic, Tokyo 173-0016, Japan; 8 Wada Pediatric Clinic, Tokyo 121-0812, Japan; 9 Suitengumaeikiiki Clinic, Tokyo 103-0014, Japan; 10 Sotobo Child Clinic, Chiba 299-4503, Japan; 11 Saitama Citizens Medical Center, Saitama 331-0054, Japan; 12 Division of Infectious Diseases and Infection Control, Tohoku Medical and Pharmaceutical University, Sendai 983-8536, Japan; 13 Nakagawa Ekimae Naika Clinic, Kanagawa 224-0001, Japan; 14 Division of Infectious Diseases, Advanced Clinical Research Center, Institute of Medical Science, University of Tokyo, Tokyo 108-8639, Japan; 15 Department of Infectious Diseases and Applied Immunology, IMSUT Hospital of the Institute of Medical Science, University of Tokyo, Tokyo 108-8639, Japan; 16 Department of Special Pathogens, International Research Center for Infectious Diseases, Institute of Medical Science, University of Tokyo, Tokyo 108-8639, Japan; 17 Research Center for Global Viral Diseases, National Center for Global Health and Medicine Research Institute, Tokyo 162-8655, Japan; 18 The University of Tokyo Pandemic Preparedness, Infection and Advanced Research Center (UTOPIA), Minato-ku, Tokyo 108-8639, Japan

**Keywords:** influenza A virus, H1N1pdm09, antigenic change, human, ferret, antigenicity

## Abstract

The influenza A(H1N1)pdm09 virus that emerged in 2009 causes seasonal epidemic worldwide. The virus acquired several amino acid substitutions that were responsible for antigenic drift until the 2018–2019 influenza season. Viruses possessing mutations in the NA and PA proteins that cause reduced susceptibility to NA inhibitors and baloxavir marboxil, respectively, have been detected after antiviral treatment, albeit infrequently. Here, we analyzed HA, NA, and PA sequences derived from A(H1N1)pdm09 viruses that were isolated during the 2018–2019 and 2019–2020 influenza seasons in Japan. We found that A(H1N1)pdm09 viruses possessing the D187A and Q189E substitutions in HA emerged and dominated during the 2019–2020 season; these substitutions in the antigenic site Sb, a high potency neutralizing antibody-eliciting site for humans, changed the antigenicity of A(H1N1)pdm09 viruses. Furthermore, we found that isolates possessing the N156K substitution, which was predicted to affect the antigenicity of A(H1N1)pdm09 virus at the laboratory level, were detected at a frequency of 1.0% in the 2018–2019 season but 10.1% in the 2019–2020 season. These findings indicate that two kinds of antigenically drifted viruses—N156K and D187A/Q189E viruses—co-circulated during the 2019–2020 influenza season in Japan.

## 1. Introduction

Influenza A virus contains eight single-stranded negative-sense RNA segments as its genome. The viral RNAs are associated with nucleoprotein (NP) and a heterotrimeric polymerase complex that consists of the polymerase basic 2 (PB2), PB1, and polymerase acidic (PA) subunits [1]. The viruses possess two surface glycoproteins, hemagglutinin (HA) and neuraminidase (NA). HA is the main target for neutralizing antibodies, which suppress virus propagation in vitro and in vivo. To escape recognition by neutralizing antibodies, the viruses gradually accumulate amino acid substitutions in their HA, resulting in an antigenic change [2]. Pandemic 2009 influenza A subtype H1N1 [A(H1N1)pdm09] virus has five major antigenic sites in its HA [3,4,5,6]. Two antigenic sites (Sa and Sb) are located near the receptor binding site (RBS) and elicit high potency neutralizing antibodies [3,4]. The antigenic site Ca, which comprises Ca1 and Ca2, spans the intermonomer interface in the HA head, and the antigenic site Cb in the HA head maps to a region close to the HA stem [3,4]. Major antigenic changes in human influenza A subtype H3N2 [A(H3N2)] viruses are predominantly caused by single amino acid substitutions at sites near the RBS [7]. Antigenic change may also be a secondary effect of amino acid substitutions that improve viral fitness for human infection [8]. Monitoring such antigenic changes is important for the selection of isolates that will be used for vaccine production.

Since the A(H1N1)pdm09 virus emerged in 2009, it has been circulating in humans as a seasonal influenza virus [9]. The antigenicity of A(H1N1)pdm09 viruses did not change until the emergence of viruses that were classified into the subclade 6B.1 during the 2015–2016 influenza season. Antiserum from infected ferrets revealed that the antigenicity of subclade 6B.1 viruses was similar to that of previous viruses, whereas human sera from healthy donors revealed the antigenic drift of subclade 6B.1 viruses, which was caused by an amino acid substitution at position 163 in antigenic site Sa [10]. During the 2018–2019 influenza season, an epidemic in European and other countries was caused by subclade 6B.1A viruses that possessed proline at position 183 (subclades 183P-1–7) in antigenic site Sb [11]. Sera from children, but not ferret antisera, showed that the antigenicity of subclade 6B.1A viruses differed from that of subclade 6B.1 viruses [11,12]. In response to these antigenic changes, the vaccine strains have been changed three times since the 2017–2018 season [13,14]. Although ferret antisera play a role as a standard in investigations of the antigenic change in seasonal influenza viruses, such antisera sometimes fail to detect antigenic changes that are revealed by human sera. Therefore, both human sera, as well as ferret antisera are important for the characterization of the antigenicity of A(H1N1)pdm09 viruses.

An NA inhibitor (NAI) that targets the sialidase activity of NA and baloxavir marboxil (BXM), which targets the cap-dependent endonuclease activity of PA, have been widely used as anti-influenza drugs, especially in Japan. After treatment, amino acid substitutions in NA and PA that reduced the susceptibility to NAI and BXM, respectively, have occasionally been detected. Since the spread of such mutant viruses is a concern [15,16,17,18,19], it is important to monitor the prevalence of such mutants routinely.

In this study, we genetically and antigenically characterized A(H1N1)pdm09 viruses isolated during the 2018–2019 and 2019–2020 influenza seasons in Japan.

## 2. Materials and Methods

### 2.1. Ethics Statement

After informed consent was obtained, respiratory specimens were collected from patients with influenza-like symptoms who visited clinics in Chiba, Kanagawa, Miyagi, Saitama, and Tokyo, Japan, by following the protocol approved by the Research Ethics Review Committee of the Institute of Medical Science, the University of Tokyo (approval number 30-77-B0304). Human blood samples were obtained from volunteers (approval numbers: 29-71-A0320 and 29-72-A0322). All experiments were performed in accordance with the University of Tokyo’s guidelines and regulations.

### 2.2. Cells

The hCK cells, which are Madin–Darby canine kidney (MDCK) cells that express high levels of human-type influenza virus receptors and low levels of avian-type virus receptors [20], were cultured in minimal essential medium (MEM) containing 5% newborn calf serum (NCS) with 2 µg mL^−1^ puromycin and 10 µg mL^−1^ blasticidin at 37 °C. MDCK cells were cultured in MEM containing 5% NCS at 37 °C.

### 2.3. Virus Isolation and Sequencing

During the 2018–2019 and 2019–2020 influenza seasons, 105 and 218 A(H1N1)pdm09 viruses, respectively, were isolated from respiratory specimens by using the hCK cells incubated at 33 °C. Viral RNA was extracted from the isolated viruses by using ISOGEN-LS (Nippon gene). The HA, NA, and PA genes were amplified by using the SuperScript III One-Step RT–PCR System with Platinum Taq DNA polymerase (Invitrogen) and the following primers: HA-U12+16F (5′-AGCAAAAGCAGGGGAAAATAAAAGCAAC-3′) and HA-U13+16R (5′-AGTAGAAACAAAGGGTGTTTTTTCTCATG-3′) for HA; NA-U12+15F (5′-AGCAAAAGCAGGAGTTCAAAATGAATC-3′) and NA-U13+17R (5′-TTATATGGTCTCGTATTAGTAGAAACAAGG-3′) for NA; and PA-U12+15F (5′-AGCAAAAGCAGGTACTGATCCAAAATG-3′) and PA-U13+20R (5′-AGTAGAAACAAGGTACTTTTTCGGACAGTATGG-3′) for PA. If no PCR products were detected, RT-PCR was repeated using the following primers: HA-U12+16F, HA-420R (5′-TTTCAAATGATGACACTGAGCTCA-3′), HA-301F (5′-CAAGCTCATGGTCCTACATTGTGG-3′), and HA-U13+16R for HA; NA-U12+15F, NA-480R (5′-GGCTCCTGTCTTTAATGGTTCCATTGG-3′), NA-301F (5′-TTAGTGGATGGGCTATATACAG-3′), and NA-U13+17R for NA; and PA-U12+15F, PA-1440R (5′-TGCATTGAGCAAGGCCGTATTTATG-3′), PA-1201F (5′-TATGACAGTGATGAGCCAGAGCCC-3′), and PA-U13+20R for PA. The PCR products were purified using the Gel Extraction Kit (Qiagen) and were Sanger sequenced using the following primers: for HA, we used primers HA-12+16F, HA-301F, HA-601F (5′-TCCTCGTGCTATGGGGCATTCACC-3′), HA-1201F (5′-CTAACAAAGTAAATTCTGTTATTG-3′), and HA-420R; for NA, we used NA-U12+15F, NA-301F, NA-601F (5′-GAATTTCTGGCCCAGACAATGGGGC-3′), NA-1081F (5′-GCAATGGTGTTTGGATAGGGAGAAC-3′), and NA-480R; and for PA, we used PA-U12+15F, PA-301F (5′-AGTATATGTAACACAACAGGGGTAG-3′), PA-601F (5′-CAGTCCGAAAGAGGCGAAGAGAC-3′), PA-901F (5′-GAAGACCCGAGTCACGAGGGGGAGGG-3′), PA-1201F, PA-1801F (5′-GAGAGCATGATTGAGGCCGAGTCTTCTG-3′), and PA-540R (5′-TTTGATTCTTGCCCTGCTCTCTTCG-3′).

### 2.4. Viruses

Seven A(H1N1)pdm09 viruses (A/Singapore/GP1908/2015, A/Tokyo/UT-WD039-0/2019, A/Tokyo/UT-BB251/2019, A/Tokyo/UT-BB262/2019, A/Tokyo/UT-GR156/2019, A/Tokyo/UT-GR253/2020, and A/Tokyo/UT-AC109/2020) were propagated in the hCK or MDCK cells with MEM containing 1 µg mL^−1^ of L-1-tosylamide-2-phenylethyl chloromethyl ketone (TPCK)-treated trypsin at 33 or 37 °C, respectively. No additional mutations were detected in these viruses before and after propagation. Virus titers (50% tissue culture infectious doses, TCID_50_) in the hCK cells were determined according to the Behrens–Karber method [21].

### 2.5. Phylogenetic Analysis

HA sequences determined in this study were aligned by using MAFFT (https://mafft.cbrc.jp/alignment/server/index.html, accessed on 23 May 2021). A phylogenetic tree based on the amino acid sequences was generated by using the Maximum Likelihood method and the Jones–Taylor–Thornton (JTT) model using MEGA11 software [22].

### 2.6. Structural Analysis

Amino acid positions were plotted to the steric structure of the HA protein based on A/California/04/2009 (PDB ID: 3ubn), as a representative of A(H1N1)pdm09 viruses, by using PyMOL.

### 2.7. Plasma Samples

Sixty-three plasma samples were obtained from vaccinees who received the 2017–2018 seasonal influenza vaccine and from patients who were infected with A(H1N1)pdm09 during the 2015–2016 and 2018–2019 seasons at 1 month after vaccination or infection. Each blood sample was collected in a vacuum tube containing EDTA-2NA. Then, plasma was separated from the blood by using a Leucosep (Greiner Bio-One, Kremsmünster, Austria) and centrifugation at 1000× *g* for 15 min at room temperature. Aliquots (of plasma samples) were transferred to new tubes and kept at −20 °C until use. Eight ferret antisera were collected from four pairs of ferrets that were infected intranasally with A/Singapore/GP1908/2015, A/Tokyo/UT-WD039-0/2019, A/Tokyo/UT-GR156/2019, or A/Tokyo/UT-GR253/2020 at 4 weeks after inoculation. Before use in the assays, the plasma samples and ferret antisera were treated with receptor-destroying enzyme II (RDE II) (Denka Seiken, Tokyo, Japan) at 37 °C for 18–20 h. RDE II was then inactivated by incubation at 56 °C for 30–60 min.

### 2.8. Hemagglutination Inhibition (HI) Assay

To remove nonspecific agglutinins, one volume of 0.5% turkey red blood cells (RBCs) was added to the ferret antisera or human plasma samples, which were then incubated for 1 h at room temperature. After centrifugation at 1200× *g* rpm for 10 min, the supernatants were collected and examined for nonspecific agglutinins. The supernatants without nonspecific agglutinins were utilized for the following test. The indicated viruses (4 HA units in 25 μL) were incubated with 25 µL of two-fold serially diluted plasma samples at room temperature for 30 min. Then, 0.5% turkey RBCs were added to the mixture and incubated for 30 min at room temperature. Reciprocal numbers of the minimum dilutions of plasma samples required to inhibit hemagglutination were used as the HI titers. Experiments were repeated three times, and representative values were selected based on the three experiments.

### 2.9. Virus Neutralization Assay

The indicated viruses (100 TCID_50_ in 50 μL) were incubated with 50 µL of two-fold serially diluted ferret antisera or human plasma samples at 33 °C for 30 min. The mixtures were then added to confluent hCK cells in 96-well microplates and incubated at 33 °C for 1 h. After the mixtures were removed, the cells were washed by MEM, incubated with MEM containing 1 µg mL^−1^ of TPCK-treated trypsin at 33 °C for 3 days, and then, they were examined for cytopathic effects. Reciprocal numbers of the minimum dilution of plasma or antiserum required to inhibit the appearance of cytopathic effects were used as the neutralization titers. Experiments were conducted in duplicate and repeated three times. For each experiment, the lower of two titer values was selected as a representative value. Then, a final neutralization titer was calculated by averaging the three selected independent values.

### 2.10. Antigenic Cartography

The neutralization and HI titers of ferret sera against virus isolates were summarized separately using antigenic cartography [23]. Briefly, antigens and sera are positioned in a Euclidean space such that the distance between a serum and an antigen reflects the log difference between their titration and the maximum titration of that serum. We ran 500 optimizations using different random starting configurations. Due to the low number of antigenic variants in these datasets, their small size, and low residual stress, we did not test higher dimensional maps.

### 2.11. Statistical Analysis

GraphPad Prism software version 9.3.0 (GraphPad Software, San Diego, CA, USA) was used to calculate *p* values. We compared HI and neutralization titers by using a nonparametric Friedman test followed by Dunn’s multiple comparisons. Differences between viruses were considered significant for *p* values < 0.05.

## 3. Results

### 3.1. Genetic Characterization of A(H1N1)pdm09 Viruses Isolated during the 2018–2019 and 2019–2020 Influenza Seasons in Japan

**Figure 1:**
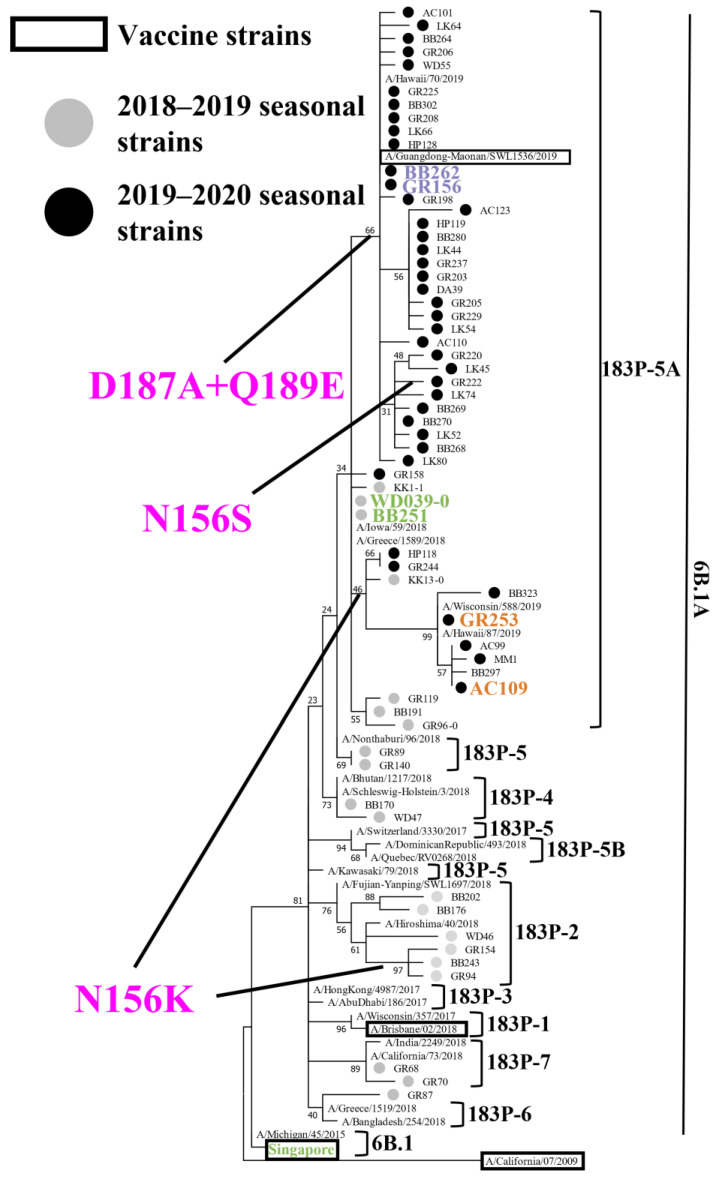
Phylogenetic analysis of HA. Phylogenetic tree based on the HA amino acid sequences of A(H1N1)pdm09 viruses, generated by using the Maximum Likelihood method and the Jones–Taylor–Thornton (JTT) model. Several HA sequences of representative isolates that we sequenced were used. Gray and black circles indicate isolates that were isolated in this study during the 2018–2019 and 2019–2020 influenza seasons, respectively. Current and previous vaccine strains are indicated by black squares. The branch bootstrap score is listed next to the corresponding subtree node. Amino acid substitutions delineating major branches are shown. A/Tokyo/UT-WD039-0/2019 (WD039-0) and A/Tokyo/UT-BB251/2019 (BB251) indicated in green; A/Tokyo/UT-BB262/2019 (BB262) and A/Tokyo/UT-GR156/2019 (GR156) indicated in purple; A/Tokyo/UT-GR253/2020 (GR253), and A/Tokyo/UT-AC109/2020 (AC109) indicated in orange; and A/Singapore/GP1908/2015 (Singapore) shown in green were used for the antigenic analysis. A/California/04/2009 is defined as an outgroup.

**Figure 2:**
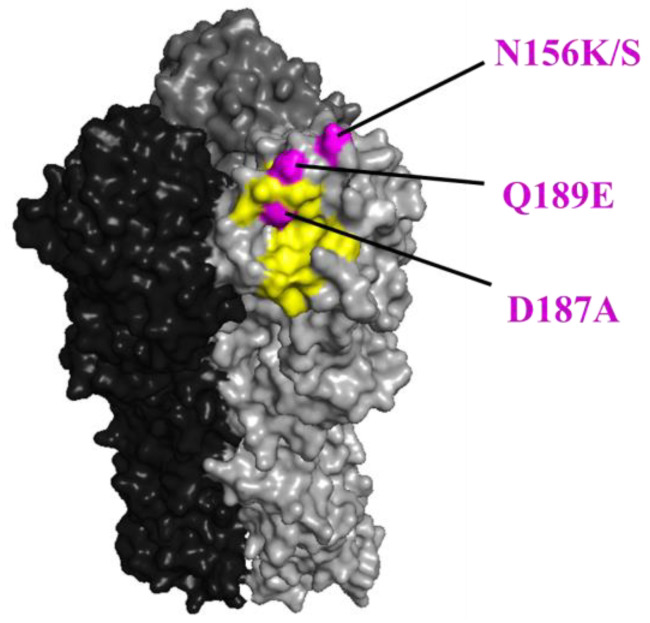
The positions of the N156K/S, D187A, and Q189E substitutions in HA are shown in magenta. The positions (H1 numbering) are shown on the HA structure of A/California/04/2009 (PDB ID: 3ubn). The amino acids that are important for receptor binding are colored yellow.

To reveal the genetic and antigenic features of the A(H1N1)pdm09 viruses that circulated during the 2018–2019 and 2019–2020 influenza seasons in Japan, we attempted to isolate influenza viruses from respiratory specimens that were collected from patients with influenza-like symptoms. During the 2018–2019 and 2019–2020 seasons, respectively, 105 and 218 A(H1N1)pdm09 viruses were isolated (Table 1). To determine the HA, NA, and PA sequences of these isolates, we performed virus RNA extraction and RT-PCR, followed by the Sanger sequencing. A phylogenetic tree was generated based on the determined HA sequences together with representative HA sequences from the Global Initiative on Sharing All Influenza Data (GISAID) database (Figure 1). The 105 viruses isolated during the 2018–2019 season were classified as follows: subclade 6B.1A (1/105); subclade 183P-2 (31/105); subclade 183P-4 (4/105); subclade 183P-5 (4/105); subclade 183P-5A (63/105); and subclade 183P-7 (2/105) (Table 1). Eight isolates of subclade 183P-2 and one subclade 183P-5A isolate possessed the N156K substitution that affected the virus antigenicity [24,25,26,27]. All 218 viruses that were isolated during the 2019–2020 season were classified as subclade 183P-5A isolates. Most of these isolates [197/218 (89.4%)] possessed the D187A and Q189E substitutions, 22 isolates (10.1%) possessed the N156K substitution, and one isolate (GR222) possessed the N156S, D187A, and Q189E substitutions (Figure 1 and Figure 2, and Table 1).

The amino acid substitutions in NA and PA that are responsible for the reduced susceptibility to NAI and BXM, respectively, were not detected in any isolates from the 2018–2019 season, whereas 1.38% (3/218) and 0.91% (2/218) of the isolates from the 2019–2020 season possessed the H275Y substitution in NA and the I38T or I38F substitution in PA, respectively. Of these three patients with the H275Y substitution in NA, two were treated with oseltamivir phosphate, and the other was treated with baloxavir marboxil (BXM), indicating that the NA-H275Y mutant after baloxavir marboxil treatment might have been transmitted from other patients. Both patients with the I38T or I38F substitution in PA were treated with baloxavir marboxil, indicating that this treatment led to the selection of these mutant viruses. These results indicated that viruses with reduced susceptibility to NAI or BXM circulated at a low frequency but did not spread widely during the 2019–2020 influenza season.

### 3.2. Antigenic Characterization of A(H1N1)pdm09 Viruses with the D187A and Q189E Substitutions

Since the N156K/S substitution in the HA of A(H1N1)pdm09 viruses has been predicted to affect antigenicity at the laboratory level [24,26], we compared the antigenicity of isolates with or without the D187A and Q189E substitutions. We selected A/Tokyo/UT-WD039-0/2019 (WD039-0) and A/Tokyo/UT-BB251/2019 (BB251) as representative isolates that lack both substitutions and A/Tokyo/UT-BB262/2019 (BB262) and A/Tokyo/UT-GR156/2019 (GR156) as representative isolates with both substitutions. The HA sequences of WD039-0 and BB251 were identical to those of BB262 and GR156 except for positions 187 and 189. We also included A/Tokyo/UT-GR253/2020 (GR253) and A/Tokyo/UT-AC109/2020 (AC109) as representative isolates possessing the N156K substitution and A/Singapore/GP1908/2015 (Singapore; subclade 6B.1), which was used for the seasonal influenza vaccine during the 2017–2018 and 2018–2019 seasons in Japan, in our antigenic testing. To examine the antigenicity of these seven isolates, we used eight antisera that were obtained from each of two ferrets infected with Singapore, WD039-0, GR156, or GR253, and 63 plasma samples that were obtained from 54 human volunteers who received the 2017–2018 seasonal influenza vaccine and nine patients who were infected with A(H1N1)pdm09 viruses in the 2015–2016 or 2018–2019 seasons, at 1 month after vaccination or infection. We used these isolates, ferret antisera, and human plasma samples in a hemagglutination inhibition (HI) assay and a virus neutralization assay.

**Figure 3:**
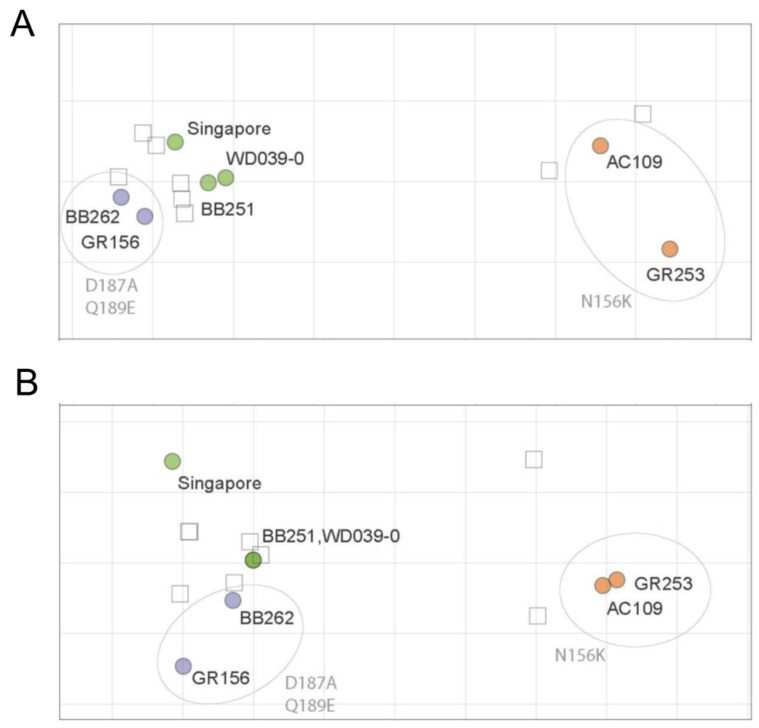
HI and neutralization antigenic maps of A(H1N1)pdm09 viruses. HI (**A**) and neutralization (**B**) titers of ferret antisera were used to construct antigenic maps. Virus names are black, whereas amino acid substitutions are grey. Viruses are abbreviated as follows: A/Singapore/GP1908/2015 (Singapore); A/Tokyo/UT-WD039-0/2019 (WD039-0); A/Tokyo/UT-BB251/2019 (BB251); A/Tokyo/UT-BB262/2019 (BB262); A/Tokyo/UT-GR156/2019 (GR156); A/Tokyo/UT-GR253/2020 (GR253); and A/Tokyo/UT-AC109/2020 (AC109). Data were analyzed by using a nonparametric Friedman test followed by Dunn’s multiple comparisons.

The ferret antisera against Singapore, WD039-0, or GR156 showed similar HI titers against Singapore, WD039-0, BB251, BB262, and GR156, but 16-times lower HI titers against GR253 and AC109 than against homologous isolates (Table 2). The ferret antisera against GR253 showed similar HI titers against GR253 and AC109 but showed four-times lower HI titers against Singapore, WD039-0, BB251, BB262, and GR156 than against homologous isolates (Table 2). The ferret antisera showed a similar pattern in the neutralization assay to that obtained in the HI assay; ferret antisera against Singapore, WD0390-0, and GR156 similarly neutralized Singapore, WD039-0, BB251, BB262, and GR156 to the homologous isolate (Table 3). The neutralization titer against GR253 and AC109 was eight times lower than that against homologous isolates. The ferret antisera against GR253 showed similar neutralization titers against GR253 and AC109, but four-times lower titers against Singapore, WD039-0, BB251, BB262, and GR156 than homologous isolates. Using these ferret HI and neutralization titers, we generated antigenic maps (Figure 3). The antigenic map of the HI titers was similar to that of the neutralization titers, demonstrating that the antigenicity of AC109 and GR253 clearly differed from that of Singapore, WD039-0, BB251, BB262, and GR156. These results indicate that the antigenicity of A(H1N1)pdm09 viruses is more affected by the N156K substitution than the D187A and Q189E substitutions.

**Figure 4:**
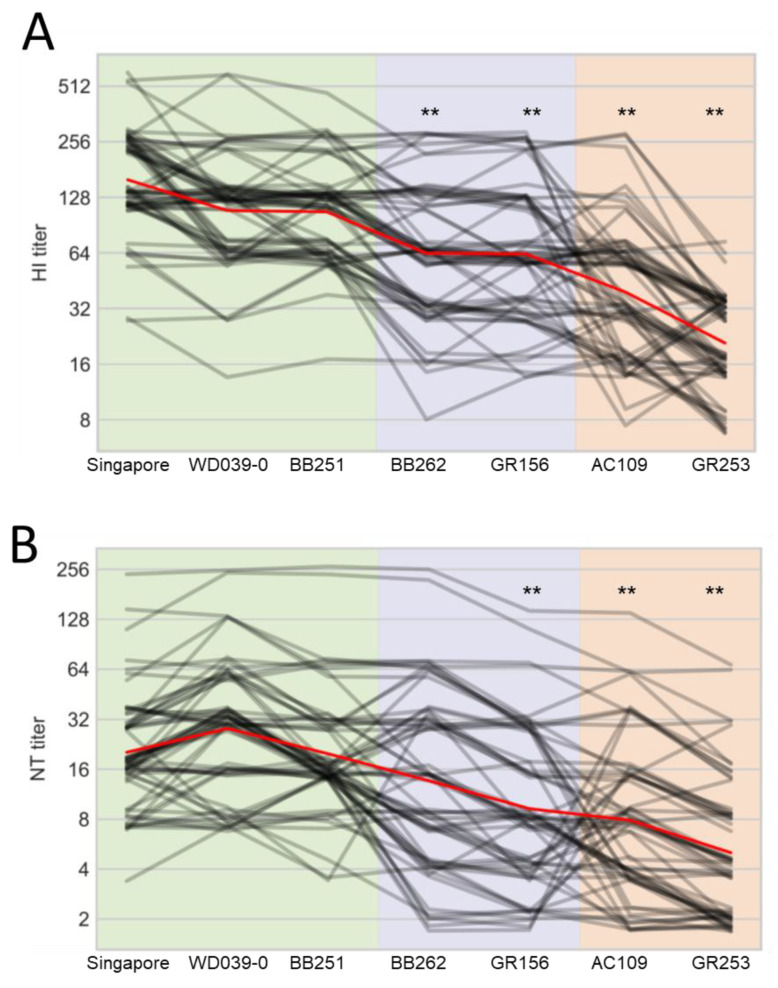
Reactivity of human plasma samples against A(H1N1)pdm09 viruses. HI (**A**) and neutralization (**B**) titers of 63 human serum samples against the indicated seven viruses are plotted as black lines; a small amount of vertical jitter has been added to prevent lines from overlapping each other. The mean of all individuals is shown in red. Antigens are grouped on the *x*-axis according to the substitutions of interest. Viruses are abbreviated as follows: A/Singapore/GP1908/2015 (Singapore); A/Tokyo/UT-WD039-0/2019 (WD039-0); A/Tokyo/UT-BB251/2019 (BB251); A/Tokyo/UT-BB262/2019 (BB262); A/Tokyo/UT-GR156/2019 (GR156); A/Tokyo/UT-GR253/2020 (GR253); and A/Tokyo/UT-AC109/2020 (AC109). ** indicates *p* < 0.01 compared with Singapore.

For the human plasma samples, the HI titers against WD039-0 and BB251 were similar to that against Singapore, whereas those against BB262, GR156, AC109, and GR253 were significantly lower than that against Singapore (Figure 4A). Neutralization titers against GR156, AC109, and GR253 were lower than that against Singapore (Figure 4B). The other three viruses showed similar antigenicity to Singapore in the neutralization assay. These results demonstrate that human plasma samples, unlike ferret antisera, distinguish the antigenicity of BB262 and GR156 from that of Singapore, WD039-0, and BB251.

When we looked at the results individually, five samples (ID 8, 29, 42, 50, and 54) showed the four-times lower HI titers against BB262 and GR156 than those against WD039-0 and BB251, whereas 23 samples (ID 4, 5, 7, 8, 9, 14, 16, 18, 19, 20, 23, 24, 27, 29, 30, 31, 33, 35, 36, 52, 54, 56, and 60) showed the four-times lower HI titers against GR253 and AC109 than those against WD039-0 and BB251 (Table S1). For the neutralization titers, 12 samples (ID 2, 8, 23, 28, 29, 30, 31, 41, 44, 49, 50, and 56) showed the four-times lower neutralization titers against BB262 and GR156 than those against WD039-0 and BB251, whereas 23 samples (ID 4, 6, 8, 9, 11, 18, 19, 23, 24, 27, 28, 29, 30, 33, 35, 36, 39, 44, 45, 47, 54, 55, and 61) showed the four-times lower neutralization titers against GR253 and AC109 than those against WD039-0 and BB251 (Table S2).

Taken together, these results demonstrate that human sera, but not ferret antisera, recognize antigenic differences depending on the D187A and Q189E substitutions in the HA of A(H1N1)pdm09 viruses.

## 4. Discussion

In this study, we determined the HA, NA, and PA sequences of 323 A(H1N1)pdm09 viruses that were isolated during the 2018–2019 and 2019–2020 influenza seasons. The HA sequence analysis revealed that A(H1N1)pdm09 viruses possessing the D187A and Q189E substitutions in HA emerged and dominated during the 2019–2020 influenza season. Of note, surveillance from 2017 to 2020 in Thailand revealed that isolates possessing the D187A and Q189E substitutions circulated during the 2019–2020 influenza season [27]. Although N-linked glycosylation at the HA head is a major driving force of antigenic change by hiding the epitopes near the glycosylation site [28,29], the D187A and Q189E substitutions do not change the glycosylation pattern. Rather, both the D187A and Q189E substitutions occur at antigenic site Sb, one of the five major antigenic sites in the HA head [3,4,5,6], where they alter the hydrophilicity and electric status. Such amino acid changes are frequently responsible for the major antigenic changes in A(H3N2) virus [7]. The amino acid substitution of D to A causes the loss of an acidic amino acid, whereas the Q to E amino acid substitution causes the acquisition of an acidic amino acid, slightly altering the charges of the amino acid positions in the antigenic site Sb of the HA head. This alteration facilitates escape from some neutralizing antibodies that recognize the antigenic site Sb without reducing the viruses’ fitness. Therefore, it is likely that the D to A and Q to E substitutions at positions 187 and 189, respectively, caused the antigenic change in A(H1N1)pdm09 virus.

Although A(H1N1)pdm09 viruses possessing the D187A and Q189E substitutions dominated during the 2019–2020 season in Japan, 22 isolates possessing the N156K substitution, which was predicted to affect the virus antigenicity [24,26], were also detected. This finding indicates that viruses possessing the N156K substitution and viruses possessing the D187A and Q189E substitutions co-circulated. The viruses possessing the N156K substitution emerged during the 2018–2019 influenza season. The detection rate of such isolates in the 2019–2020 season was 10.1%, increasing from 1.0% during the previous season. According to the sequences registered in GISAID, the detection rate has gradually increased (0.17%, 2.10%, and 21.98% in the 2017–2018, 2018–2019, and 2019–2020 seasons, respectively), and according to the Nextstrain project (https://nextstrain.org/, accessed on 29 December 2020) [30], in 2021 and 2022, A(H1N1)pdm09 viruses possessing the N156K substitution (6B.1A.5a.2) co-circulated with viruses possessing the D187A and Q189E substitutions (6B.1A.5a.1). Based on the increase in detection rate, we speculate that these N156K-substituted viruses were either introduced into Japan or emerged due to antigenic drift.

The antigenic change caused by the D187A and Q189E substitutions or the N156K substitution likely reduced vaccine efficacy in the 2018–2019 and 2019–2020 seasons. Isolates without these substitutions may have transmitted less efficiently among vaccinees, whereas the antigenically changed isolates likely efficiently transmit even among vaccinees. Therefore, the influenza virus vaccine strains should be appropriately updated based on the antigenicity of the currently circulating virus strains.

Amino acid substitutions in NA and PA that reduce virus susceptibility to NAI and BXM, respectively, have occasionally been detected, and the spread of such mutant viruses is a concern [15,16,17,18,19]. The WHO GISRS Expert Working Group for Surveillance of Antiviral Susceptibility (WHO-AVWG) conducted global surveillance to determine the NAI and BXM susceptibility of influenza viruses during the 2012–2013 and 2017–2018 seasons, respectively [31,32]. It reported detection rates of A(H1N1)pdm09 viruses with mutant NA that ranged from 0.9% to 1.8% from 2012–2013 to the 2017–2018 season [31,32,33,34,35,36]. The detection rate of viruses with mutant PA was 0.2% in the 2017–2018 season [32]. In our study, viruses with such mutations were not detected among the isolates from the 2018–2019 season, whereas 1.38% (3/218) and 0.92% (2/218) of isolates from the 2019–2020 season possessed the H275Y substitution in NA and the I38T or I38F substitution in PA, respectively. Although the detection rate was not drastically increased from that reported previously, continuous monitoring of viruses with reduced susceptibility to NAI and/or BXM is warranted for the early detection of such mutant viruses.

In our study, ferret antisera failed to recognize the antigenic difference due to the D187A and Q189E substitutions. A similar phenomenon has been observed with recent human A(H1N1)pdm09 and H3N2 viruses and might be explained by the fact that immunologically naïve ferrets have different immune responses to infection with influenza virus compared to humans who have complex histories of infection and vaccination [37]. Moreover, ferret antisera predominantly recognize the antigenic site Sa, whereas humans show a different immunodominance pattern, in which sites Sb and Sa are immunodominant [38]. Since the D187A and Q189E substitutions map to the antigenic site Sb, ferrets may not recognize these changes well. Therefore, we need to consider animal species-specific immune responses that could affect recognition patterns and recognize that serum samples derived from several animals, including humans, may be required to characterize antigenicity correctly.

In summary, isolates possessing the D187A and Q189E substitutions emerged and dominated during the 2019–2020 season, and their antigenicity differed from that of isolates without these substitutions. We need to closely monitor the antigenic drift of these viruses.

## Figures and Tables

**Table 1:** Phylogenetic classification based on the HA of A(H1N1)pdm09 viruses isolated during the 2018–2019 and 2019–2020 influenza seasons.

Influenza Season	Amino Acid Substitution in HA	Subclade						Total
		6B.1A	183P-2	183P-4	183P-5	183P-5A	183P-7	
2018–2019	N156K	0	8 (7.6%)	0	0	1 (1.0%)	0	9 (8.6%)
	D187A and Q189E	0	0	0	0	0	0	0
	N156S, D187A, and Q189E	0	0	0	0	0	0	0
	Others	1 (1.0%)	23 (21.9%)	4 (3.8%)	4 (3.8%)	62 (59.0%)	2 (1.9%)	96 (91.4%)
	Total	1 (1.0%)	31 (29.5%)	4 (3.8%)	4 (3.8%)	63 (60.0%)	2 (1.9%)	105
2019–2020	N156K	0	0	0	0	22 (10.1%)	0	22 (10.1%)
	D187A and Q189E	0	0	0	0	195 (89.4%)	0	195 (89.4%)
	N156S, D187A, and Q189E	0	0	0	0	1 (0.5%)	0	1 (0.5%)
	Others	0	0	0	0	0	0	0
	Total	0	0	0	0	218 (100%)	0	218

**Table 2:** Hemagglutination inhibition (HI) titers of ferret sera against A(H1N1)pdm09 viruses.

Vaccine Strain:		Ferret ID Immunized against:							
		Singapore ^a^		WD039-0 ^b^		GR156 ^e^		GR253 ^f^	
		111	112	121	122	131	132	141	142
2017–2019 season	Singapore ^a^	**2048**	**1024**	1024	2048	1024	4096	64	64
2018–2019 season	WD039-0 ^b^	1024	1024	**2048**	**2048**	1024	2048	128	128
	BB251 ^c^	2048	1024	2048	4096	1024	2048	128	128
2019–2020 season	BB262 ^d^	1024	1024	2048	2048	2048	2048	16	32
	GR156 ^e^	1024	1024	2048	4096	**1024**	**2048**	16	32
	GR253 ^f^	16	16	32	64	16	32	**512**	**1024**
	AC109 ^g^	32	32	64	128	32	64	1024	4096

^a^ A/Singapore/GP1908/2015, ^b^ A/Tokyo/UT-WD039-0/2019, ^c^ A/Tokyo/UT-BB251/2019, ^d^ A/Tokyo/UT-BB262/2019, ^e^ A/Tokyo/UT-GR156/2019, ^f^ A/Tokyo/UT-GR253/2020, and ^g^ A/Tokyo/UT-AC109/2020 were used. Boldface type indicates homologous combinations.

**Table 3:** Neutralization titers of ferret sera against A(H1N1)pdm09 viruses.

Vaccine Strain:		Ferret ID Immunized against:							
		Singapore ^a^		WD039-0 ^b^		GR156 ^e^		GR253 ^f^	
		111	112	121	122	131	132	141	142
2017–2019 season	Singapore ^a^	**64**	**64**	64	64	32	32	4	<4
2018–2019 season	WD039-0 ^b^	64	64	**128**	**256**	64	128	8	8
	BB251 ^c^	64	64	128	256	64	128	8	8
2019–2020 season	BB262 ^d^	64	64	64	128	64	128	4	8
	GR156 ^e^	32	32	32	64	**64**	**64**	<4	4
	GR253 ^f^	<4	<4	4	8	<4	<4	**32**	**64**
	AC109 ^g^	<4	<4	4	8	<4	4	32	64

^a^ A/Singapore/GP1908/2015, ^b^ A/Tokyo/UT-WD039-0/2019, ^c^ A/Tokyo/UT-BB251/2019, ^d^ A/Tokyo/UT-BB262/2019, ^e^ A/Tokyo/UT-GR156/2019, ^f^ A/Tokyo/UT-GR253/2020, and ^g^ A/Tokyo/UT-AC109/2020 were used. Boldface type indicates homologous combinations.

## Data Availability

The data presented in this study are available in the main text and Supplementary Materials.

## Supplementary Materials

The following supporting information can be downloaded at: https://www.mdpi.com/article/10.3390/v15020535/s1, Table S1: HI titers of human sera against A(H1N1)pdm09 viruses; Table S2: Neutralization titers of human sera against A(H1N1)pdm09 viruses.
