# Supplementary material for: Characterization of Influenza A(H1N1)pdm09 Viruses Isolated in the 2018–2019 and 2019–2020 Influenza Seasons in Japan
Source: Viruses. 2023 Feb 14;15(2):535. doi: 10.3390/v15020535 (PMC9968111; doi:10.3390/v15020535)
Supplement: Supplementary file 1 [file viruses-15-00535-s001.zip › viruses-2180969-supplementary.pdf]

Supplementary Table S1. HI titers of human sera against A(H1N1)pdm09 viruses.

Patient ID	Age	HI titer against:						
		Vaccine strain in 2017–2019 season	2018–2019 season			2019–2020 season		
		Singapore [a]	WD039-0 [b]	BB251 [c]	BB262 [d]	GR156 [e]	GR253 [f]	AC109 [g]
1	20	128	256	256	128	128	32	128
2	22	256	64	64	32	32	32	128
3	23	256	512	512	256	256	64	256
4	24	256	256	128	256	256	8	16
5	24	256	256	256	64	128	32	64
6	24	512	512	256	256	256	64	256
7	25	128	256	256	128	256	32	64
**8**	**25**	**256**	**128**	**64**	**16**	**16**	**16**	**16**
9	25	128	128	128	128	128	16	32
10	25	256	256	128	256	256	32	64
11	25	32	32	64	64	32	8	32
12	26	256	128	128	64	64	32	64
13	26	128	64	128	128	128	32	64
14	27	256	64	64	32	32	16	16
15	28	128	128	128	64	64	32	64
16	29	512	128	128	64	64	16	32
17	32	128	128	128	64	64	32	64
18	33	256	128	128	128	128	16	16
19	33	128	128	128	128	128	16	32
20	33	256	128	128	64	64	16	32
21	34	128	64	128	64	64	16	64
22	35	256	128	128	128	64	32	64
23	35	256	128	128	64	64	16	8
24	36	256	128	128	128	128	16	8
25	37	128	128	128	64	64	32	64
26	37	256	128	128	64	64	32	64
27	38	128	128	128	64	64	8	32
28	38	256	128	128	32	64	16	64
**29**	**38**	**64**	**64**	**64**	**8**	**16**	**8**	**16**
30	38	256	64	64	32	32	16	16
31	39	128	256	256	128	64	32	64

32	39	32	16	16	16	16	8	32
33	40	128	128	128	128	128	16	32
34	40	256	64	64	32	32	16	32
35	41	64	64	128	64	64	8	16
36	41	256	64	64	32	32	8	16
37	41	64	32	64	32	32	8	32
38	42	256	128	128	128	128	32	64
39	42	128	64	64	32	32	16	32
40	43	256	64	64	64	64	32	64
41	43	128	64	64	16	32	32	64
**42**	**43**	**128**	**64**	**64**	**16**	**16**	**32**	**16**
43	44	128	128	64	64	64	32	64
44	44	128	64	64	32	32	32	16
45	44	128	128	128	64	64	16	64
46	44	128	128	64	32	32	32	16
47	45	64	64	64	32	64	32	16
48	45	256	64	64	64	32	32	32
49	46	256	128	256	64	64	32	128
**50**	**46**	**128**	**128**	**64**	**16**	**16**	**16**	**32**
51	47	64	32	32	32	32	8	16
52	48	512	256	256	256	256	32	16
53	49	128	128	128	64	64	16	64
**54**	**51**	**256**	**128**	**128**	**32**	**32**	**16**	**16**
55	52	128	64	64	128	128	16	32
56	53	64	64	64	32	16	8	16
57	53	128	128	128	64	64	64	64
58	54	256	256	256	256	256	32	256
59	55	128	128	256	128	128	32	128
60	60	256	256	256	256	256	16	32
61	61	128	128	64	64	64	32	64
62	62	128	128	128	128	128	32	128
63	76	128	64	64	32	32	16	32

[a]A/Singapore/GP1908/2015, [b]A/Tokyo/UT-WD039-0/2019, [c]A/Tokyo/UT-BB251/2019, [d]A/Tokyo/UT-BB262/2019, [e]A/Tokyo/UT-GR156/2019, [f]A/Tokyo/UT-GR253/2020, and [g]A/Tokyo/UT-AC109/2020 were used.

Supplementary Table S2. Neutralization titers of human sera against A(H1N1)pdm09 viruses.

Patient ID	Age	Neutralization titer against:						
		Vaccine strain in 2017–2019 season	2018–2019 season			2019–2020 season		
		Singapore [a]	WD039-0 [b]	BB251 [c]	BB262 [d]	GR156 [e]	GR253 [f]	AC109 [g]
1	20	64	64	64	64	32	16	32
2	22	8	16	16	<4	<4	16	32
3	23	256	256	256	256	128	64	128
4	24	16	32	16	32	32	<4	<4
5	24	32	64	32	32	16	8	16
6	24	128	256	256	256	128	64	64
7	25	64	128	64	64	32	32	32
8	25	16	32	16	<4	<4	<4	<4
9	25	32	64	32	64	32	<4	4
10	25	32	128	64	64	64	16	32
11	25	16	16	16	8	8	<4	4
12	26	16	32	32	16	16	8	16
13	26	16	64	16	32	32	8	16
14	27	8	8	8	4	4	<4	4
15	28	16	32	32	32	16	8	16
16	29	8	16	16	16	8	NT	NT
17	32	32	64	32	16	8	8	16
18	33	16	32	16	64	32	<4	<4
19	33	32	64	32	64	32	4	8
20	33	16	32	16	16	8	4	8
21	34	16	32	16	8	8	4	8
22	35	8	32	16	8	4	4	8
23	35	16	32	16	<4	<4	<4	<4
24	36	16	32	16	32	16	<4	<4
25	37	32	32	16	16	8	4	8
26	37	16	16	16	16	8	4	8
27	38	32	64	16	8	8	<4	4
28	38	16	32	32	4	<4	8	8
29	38	8	32	16	4	<4	<4	4
30	38	8	16	16	4	4	<4	4

31	39	128	128	64	16	4	16	32
32	39	4	8	4	4	4	<4	8
33	40	16	32	32	32	32	4	4
34	40	16	8	16	4	4	4	8
35	41	32	32	16	4	8	<4	4
36	41	16	16	16	8	8	<4	4
37	41	8	8	8	8	8	4	4
38	42	64	64	64	64	32	32	16
39	42	16	16	16	8	8	<4	4
40	43	16	32	16	8	4	8	16
41	43	32	32	16	<4	<4	16	8
42	43	8	8	4	<4	<4	<4	<4
43	44	32	32	16	32	16	8	8
44	44	16	32	16	4	4	<4	<4
45	44	8	8	8	16	8	<4	<4
46	44	16	8	16	8	4	<4	4
47	45	8	8	16	8	4	<4	<4
48	45	8	8	8	8	4	4	4
49	46	32	32	32	8	4	8	16
50	46	16	32	16	<4	<4	8	16
51	47	8	8	8	8	8	4	4
52	48	32	64	32	32	32	NT	NT
53	49	16	64	32	32	16	8	16
54	51	16	8	16	4	<4	<4	<4
55	52	16	16	16	32	32	<4	4
56	53	16	16	16	4	4	4	8
57	53	32	32	16	16	8	4	8
58	54	64	64	64	64	64	32	64
59	55	32	64	64	64	32	16	32
60	60	16	8	4	32	8	4	8
61	61	32	32	32	32	16	4	8
62	62	32	32	16	32	32	16	64
63	76	32	8	8	4	<4	NT	NT

[a]A/Singapore/GP1908/2015, [b]A/Tokyo/UT-WD039-0/2019, [c]A/Tokyo/UT-BB251/2019, [d]A/Tokyo/UT-BB262/2019, [e]A/Tokyo/UT-GR156/2019, [f]A/Tokyo/UT-GR253/2020, and [g]A/Tokyo/UT-AC109/2020 were used. NT, not tested.
